# Efficacy and Safety of Dual vs Single Antibiotic-Loaded Cement in Bone Fracture Management: A Systematic Review and Meta-Analysis

**DOI:** 10.7759/cureus.75208

**Published:** 2024-12-06

**Authors:** Elsiddig A Ahmed, Khalid Muharib R. Alruwaili, Abdulmajeed Abdulhamid F. Alanazi, Abdulelah Alruwaili, Abdulaziz Talal M. Alruwaili

**Affiliations:** 1 Orthopedics and Traumatology, Prince Mutaib bin Abdulaziz Hospital, Sakaka, SAU; 2 Orthopedics and Traumatology Surgery, Prince Mutaib bin Abdulaziz Hospital, Sakaka, SAU; 3 Medical College, Jouf University, Sakaka, SAU

**Keywords:** arthroplasty, dual antibiotic-loaded cement, prosthetic joint infections (pji), single antibiotic-loaded cement, surgical site infection(ssi)

## Abstract

Bone fractures often require arthroplasty, which carries the risk of surgical site infections (SSIs) and prosthetic joint infections (PJIs). Antibiotic-loaded bone cement (ALBC) is commonly used to reduce these risks. Dual antibiotic-loaded cement (DALC) has been proposed as a more effective option compared to single antibiotic-loaded cement (SALC). This systematic review and meta-analysis aimed to compare the efficacy and safety of DALC and SALC in preventing infections and related outcomes in arthroplasty. We conducted a systematic review and meta-analysis comparing DALC and SALC in patients undergoing hip or knee arthroplasty for fractures. The primary outcome was infection rate (SSI and PJI), with secondary outcomes including re-revision rates and mortality. Databases searched included PubMed, Cochrane Library, Scopus, and Google Scholar. Data synthesis was performed using Review Manager Software (RevMan 5.4, Cochrane Methods, London, UK), and odds ratios (OR) with 95% confidence intervals (CI) were calculated.

Nine studies comprising 55,672 patients were included. Eight studies focused on hip arthroplasty, and four included knee surgeries. In hip arthroplasty, DALC significantly reduced infection rates compared to SALC (OR, 0.64; 95% CI, 0.49 to 0.83; P = 0.001), with moderate heterogeneity (I² = 52%). However, no significant difference was found in knee arthroplasty (OR, 1.21; 95% CI, 0.87 to 1.70; P = 0.26). Overall, DALC showed a significant reduction in infection rates (OR, 0.81; 95% CI, 0.66 to 1.00; P = 0.05). DALC also significantly reduced deep surgical site infections in hip surgeries (OR, 0.46; 95% CI, 0.33 to 0.66; P < 0.001). No significant differences were observed in re-revision rates for either hip or knee arthroplasty. Mortality rates were also not significantly different between DALC and SALC. DALC appears to reduce infection rates, particularly in hip arthroplasty, compared to SALC. However, no significant differences were found in re-revision or mortality rates. These findings suggest that DALC may offer better prophylaxis in hip surgeries, but further research is needed to explore its broader benefits and cost-effectiveness.

## Introduction and background

Bone fractures represent a significant global health concern, affecting millions annually. While surgical interventions like arthroplasty are crucial for restoring function and improving quality of life, they carry the risk of periprosthetic joint infections (PJIs) and surgical site infections (SSI). These infections can lead to severe consequences for patients, including increased morbidity, mortality, extended hospital stays, and a heavy financial burden on healthcare systems [1, 2]. This issue is particularly pressing in hip hemiarthroplasties, a common procedure for treating intracapsular femoral neck fractures, a frequently encountered injury in elderly individuals with osteoporosis [3,4].

Antibiotic-loaded bone cement (ALBC), traditionally with gentamicin, has long been used to minimize infection risk in arthroplasty. However, there is ongoing debate regarding the ideal type and application of ALBC, especially for hemiarthroplasties [5]. Dual antibiotic-loaded bone cement (DALBC), containing both gentamicin and clindamycin, has emerged as a promising solution to tackle persistent surgical site infections. DALBC's potential stems from the synergistic effect of the two antibiotics, potentially providing enhanced antimicrobial effectiveness and broader spectrum coverage compared to single antibiotic-loaded cement [6-8].

Gentamicin, a potent bactericidal antibiotic, targets numerous Gram-positive and Gram-negative bacteria often implicated in PJIs. Clindamycin, on the other hand, acts as a bacteriostatic agent, effective against Gram-positive bacteria and anaerobic pathogens. This combination aims to address a wider range of potential infectious agents, potentially bolstering the prophylactic effect [9].

However, despite promising in vitro data suggesting increased antimicrobial activity and biofilm inhibition with DALBC, real-world clinical evidence has been limited and sometimes contradictory [8-11]. While several observational studies indicate the benefit of DALBC in lowering SSIs following hip hemiarthroplasty for femoral neck fractures, concerns remain regarding its cost-effectiveness and the potential to fuel antibiotic resistance [7, 12].

This systematic review and meta-analysis endeavors to comprehensively assess the current evidence from randomized controlled trials, quasi-randomized studies, and retrospective analyses to thoroughly evaluate the efficacy and safety of DALBC in managing bone fractures [13-15].

We analyzed the data to determine if DALBC significantly reduces the risk of deep SSIs and overall SSIs in patients undergoing hip hemiarthroplasty for femoral neck fractures or in revision of these surgeries compared to single antibiotic-loaded cement.

By synthesizing data from various studies and using robust meta-analytical methods, this review aims to provide a clear picture of DALBC's clinical effectiveness and safety. The findings will be valuable for clinicians, impacting treatment guidelines and ultimately enhancing outcomes for patients with bone fractures. This analysis will contribute to the ongoing discussion about the best strategies for infection prevention and shed light on the potential of DALBC to significantly improve the management of bone fractures.

## Review

Methods

Eligibility Criteria

Our inclusion criteria were patients with hip or knee joint fractures who had undergone any type of arthroplasty whether in primary operation or subsequent revisions. For the intervention group, we included patients who were treated with dual antibiotic-loaded cement. The control group was those who were treated with single antibiotic-loaded cement or other alternative treatments. Our primary outcome was the infection rate, whether surgical site infection or prosthetic joint infection. Secondary outcomes include the re-revision rate and mortality. We included randomized controlled trials (RCTs), cohort studies, and observational studies.

Information Sources

We conducted a comprehensive database search in PubMed, Cochrane Library, Scopus, and Google Scholar for relevant articles. Our search strategy was “Dual (Antibiotic OR Clindamycin OR Gentamicin OR Erythromycin OR Colistin OR (Antibiotic-Loaded)) AND Cement.” This broad search strategy allowed us to identify the maximum number of articles.

Study Selection

**Figure 1:**
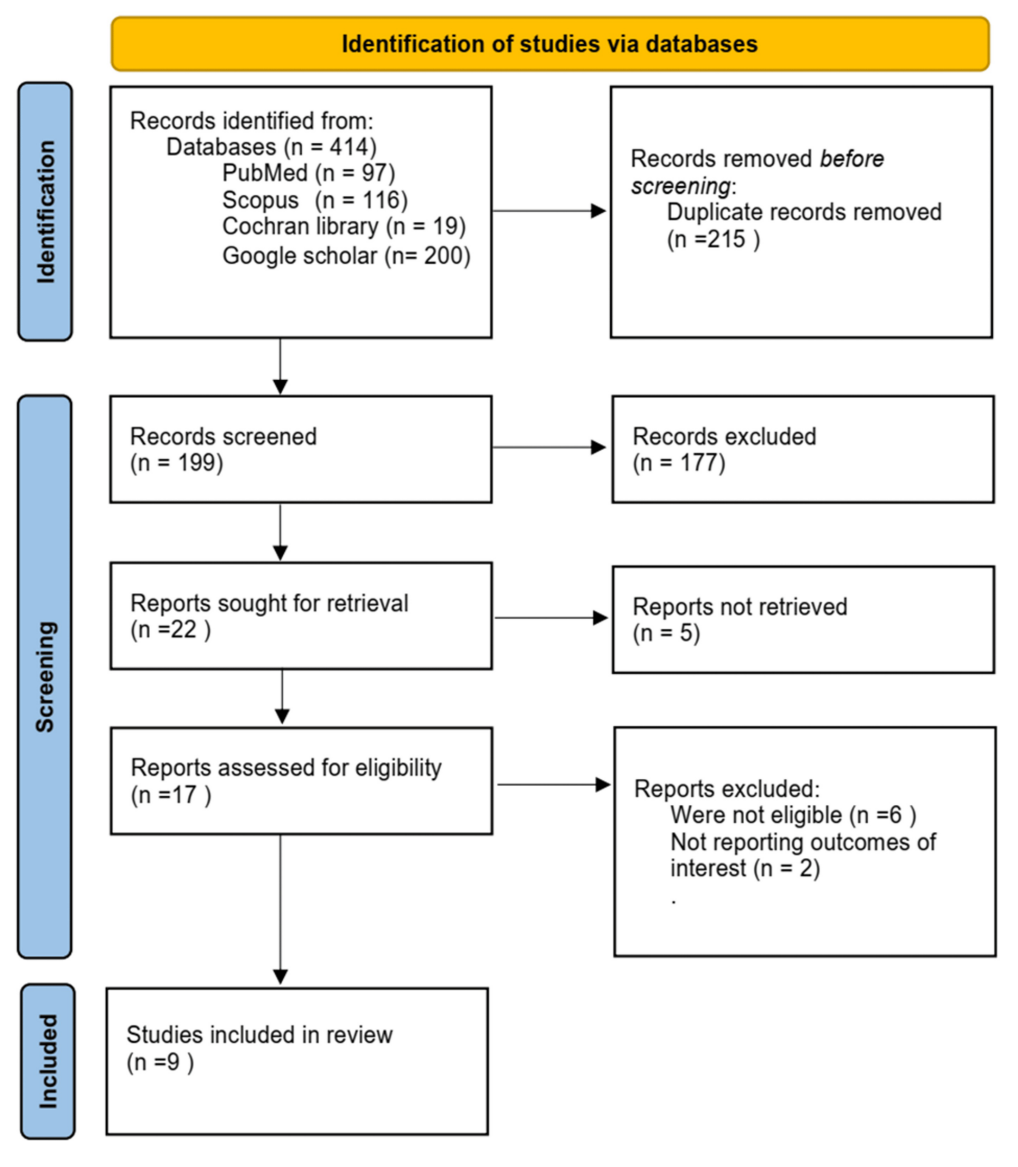
PRISMA flow diagram representing the selection process of the included papers in our systematic review. PRISMA: Preferred Reporting Items for Systematic Reviews and Meta-Analyses

The records from all the databases were uploaded to the Endnote reference manager. Then duplicates were removed. Titles and abstracts were screened by two independent reviewers to identify relevant studies. The final records from the title and abstract screening were subject to full-text screening according to the eligibility criteria. Studies were included if they met the predefined eligibility criteria. Exclusions were made based on irrelevance or lack of sufficient data (Figure 1).

Data Extraction

The data extraction sheet was designed using Google Spreadsheets (Alphabet Inc., Mountainview, USA). We extracted data on the study's characteristics, the patient's baseline characteristics, and the predefined outcomes. 

Data Synthesis

In this systematic review, we used the Review Manager Software Package (RevMan Version 5.4, Cochrane Methods, London, UK) to conduct the meta-analysis and generate the forest plots. The overall effect was calculated using the odds ratios, mean difference, and standardized mean difference depending on the outcome characteristics. We used a 95% confidence interval (95% CI), with p-values less than 0.05 considered significant. The statistical heterogeneity among the individual studies and the subgroups was evaluated based on the Cochrane Q test and the I² index. Statistical heterogeneity was confirmed if I² was >50% or P<0.10. The fixed effect model was used as the default to calculate the pooled effect. 

Results

We included nine studies [3,7,8,12,16-20] with a total of 55,672 patients in this systematic review. Six were retrospective cohort studies, one observational cohort study, a randomized controlled trial, and one quasi-randomized study. The majority of the studies included patients with a mean age above 60 years. These studies examined the efficacy of dual antibiotic-loaded cement (DALC) versus single antibiotic-loaded cement (SALC) in managing infections, such as surgical site infections (SSI) and prosthetic joint infections (PJI). Eight studies evaluate the use of DALC in hip joint surgeries. While four evaluate the knee joint. Three studies include both hip and knee joints (Table 1). 

**Table 1:** Characteristics of the Included Studies. RCT: randomized clinical trial. RC: retrospective cohort. OC: observational cohort. DALC: dual antibiotic-loaded cement. SALC: single antibiotic-loaded cement. SD: standard deviation.

Study ID	Design	Joint	Intervention	Control	Number of patients			Age (mean, SD)	
					Total	DALC	SALC	DALC	SALC
										Agni NR et al. (2023) [16]	RCT	Hip	dual-antibiotic loaded cement	single-antibiotic loaded cement	4936	2483	2453	83·9 (7·4)	83·8 (7·7)
Bos K et al. (2022) [17]	RC	Knee	Dual Antibiotic-Loaded Bone Cement	Single Antibiotic-Loaded Bone Cement	7124	1831	5293	-	-
		Hip	Dual Antibiotic-Loaded Bone Cement	Single Antibiotic-Loaded Bone Cement	2529	855	1674		
Bos PK et al. (2023) [18]	OC	Hip	dual antibiotic-loaded bone cement (ALBC)	Single Antibiotic-Loaded Bone Cement	2529	860	1669	69.5 (12)	71.2 (11)
		Knee	dual antibiotic-loaded bone cement (ALBC)	Single Antibiotic-Loaded Bone Cement	7124	1854	5270	65.2 (9.5)	66.3 (9.4)
Hamoudi C et al. (2024) [19]	RC	Hip	high-dose gentamicin and clindamycin	anti‑ biotic-loaded bone cement (ALBC) with gentamicin only	142	84	58	71 (11)	66 (11)
		Knee	high-dose gentamicin and clindamycin	anti‑ biotic-loaded bone cement (ALBC) with gentamicin only	148	61	87		
Sanz-Ruiz P et al. (2020) [8]	RC	Knee	high dose dual antibiotic loaded cement (DALBC) COPAL G+C	low dose single antibiotic loaded cement (SALBC) PALACOS R+G	246	103	143	78.5 (6.77)	79.3 (4.18)
Savage P et al. (2019) [20]	RC	Hip	dual antibiotic impregnated cement	single antibiotic impregnated cement.	206	98	108	84 (7.27)	83 (8.95)
Sprowson AP et al. (2016) [12]	quasi-randomised study	Hip	high dose dual-antibiotic impregnated cement	low dose single-antibiotic impregnated cement	848	400	448	82.96 (7.48)	82.34 (7.69)
Szymski D et al. (2023) [3]	RC	Hip	Dual Antibiotic-LoadedBone Cement	Single Antibiotic-Loaded Bone Cement	1,054	498	556	82.1 (8.28)	82.79 (7.93)
Tyas B et al. (2018) [7]	RC	Hip	High dose dual-antibiotic cement (HDDAC)	Low dose single-antibiotic cement (LDSAC)	1941	1260	681	-	-

Infection (SSI and PJI)

**Figure 2:**
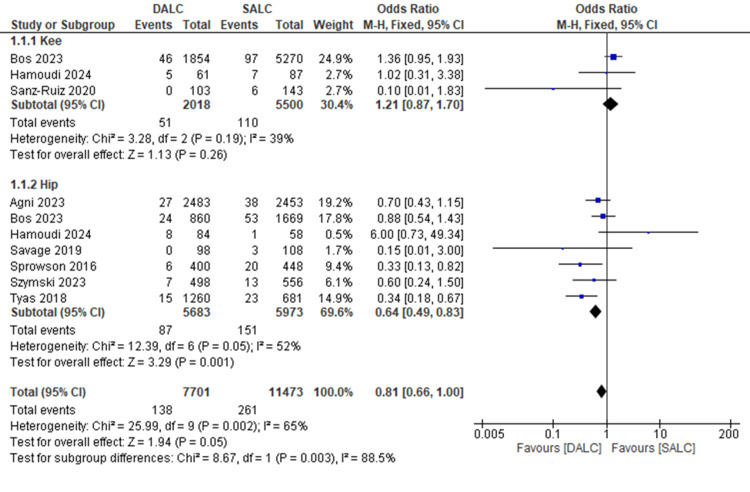
The prophylactic effect of dual antibiotics loaded cement (DALC) versus single antibiotic-loaded cement (SALC) on the rates of surgical site infections (SSI) and prosthetic joint infections (PJI). Agni 2023 [16], Bos 2023 [18], Hamoudi 2024 [19], Sanz-Ruiz 2020 [8], Savage 2019 [20], Sprowson 2016 [12], Szymski 2023 [3], Tyas 2018 [7]

We evaluated the prophylactic effect of dual antibiotic-loaded cement (DALC) versus single antibiotic-loaded cement (SALC) on the rates of surgical site infections (SSI) and prosthetic joint infections (PJI). In the knee subgroup, the odds ratio (OR) was 1.21 (95% CI (0.87, 1.70), P = 0.26), indicating no significant difference between DALC and SALC in reducing infection rates. There was moderate heterogeneity (I² = 39%). In the hip subgroup, DALC significantly reduced infection rates compared to SALC (OR = 0.64, 95% CI (0.49, 0.83), P = 0.001). Also, there was moderate heterogeneity (I² = 52%). Overall, there was a significant difference between SALC and DALC in reducing SSI and PJI rates (OR = 0.81, 95% CI (0.66, 1.00), P = 0.05). The overall heterogeneity was moderate (I² = 65%). Subgroup analysis also showed significant heterogeneity between knee and hip outcomes (I² = 88.5%, P = 0.003) (Figure 2).

Deep Surgical Infection Rate

**Figure 3:**
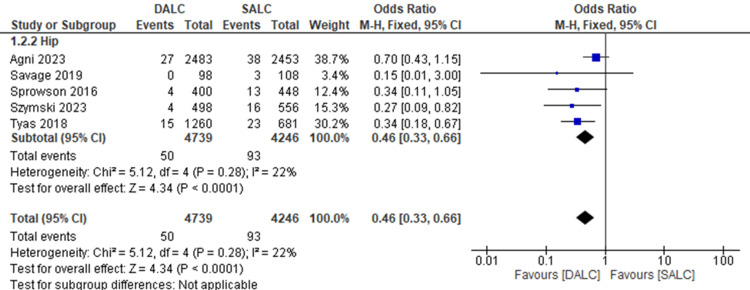
The prophylactic effects of dual antibiotics loaded cement (DALC) versus single antibiotic-loaded cement (SALC) on the deep surgical site infections rate. Agni 2023 [16], Savage 2019 [20], Sprowson 2016 [12], Szymski 2023 [3], Tyas 2018 [7]

Deep surgical site infections were reported in five studies on hip joints. DALC significantly reduced deep surgical site infection rates compared to SALC (OR = 0.46, 95% CI (0.33, 0.66), P < 0.001). There was low heterogeneity (I² = 22%) (Figure 3).

Re-Revision Rate

**Figure 4:**
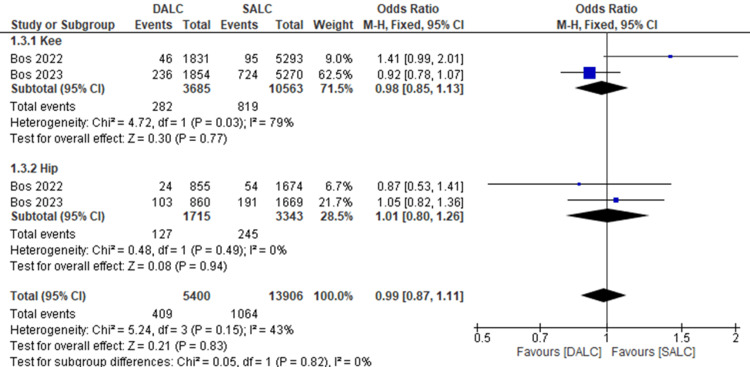
Re-revision rate of dual antibiotics loaded cement (DALC) versus single antibiotic-loaded cement (SALC). Bos 2022 [17], Bos 2023 [18].

Re-revision rate was reported in only two studies for each joint. In the knee subgroup, the odds ratio (OR) was 0.98 (95% CI (0.85, 1.13), P = 0.77), indicating no significant difference between DALC and SALC in reducing re-revision rates. There was high heterogeneity (I² = 79%) within the subgroup. Also, in the hip subgroup, DALC did not reduce re-revision rates significantly compared to SALC (OR = 1.01, 95% CI (0.80, 1.26), P = 0.94). There was no heterogeneity (I² = 0%). Overall, there was no significant difference between DALC and SALC in reducing re-revision rates (OR = 0.99, 95% CI (0.87, 1.11), P = 0.83). The overall heterogeneity was moderate (I² = 43%). Subgroup analysis showed no significant heterogeneity between knee and hip outcomes (I² = 0%, P = 0.82) (Figure 4).

Mortality

**Figure 5:**
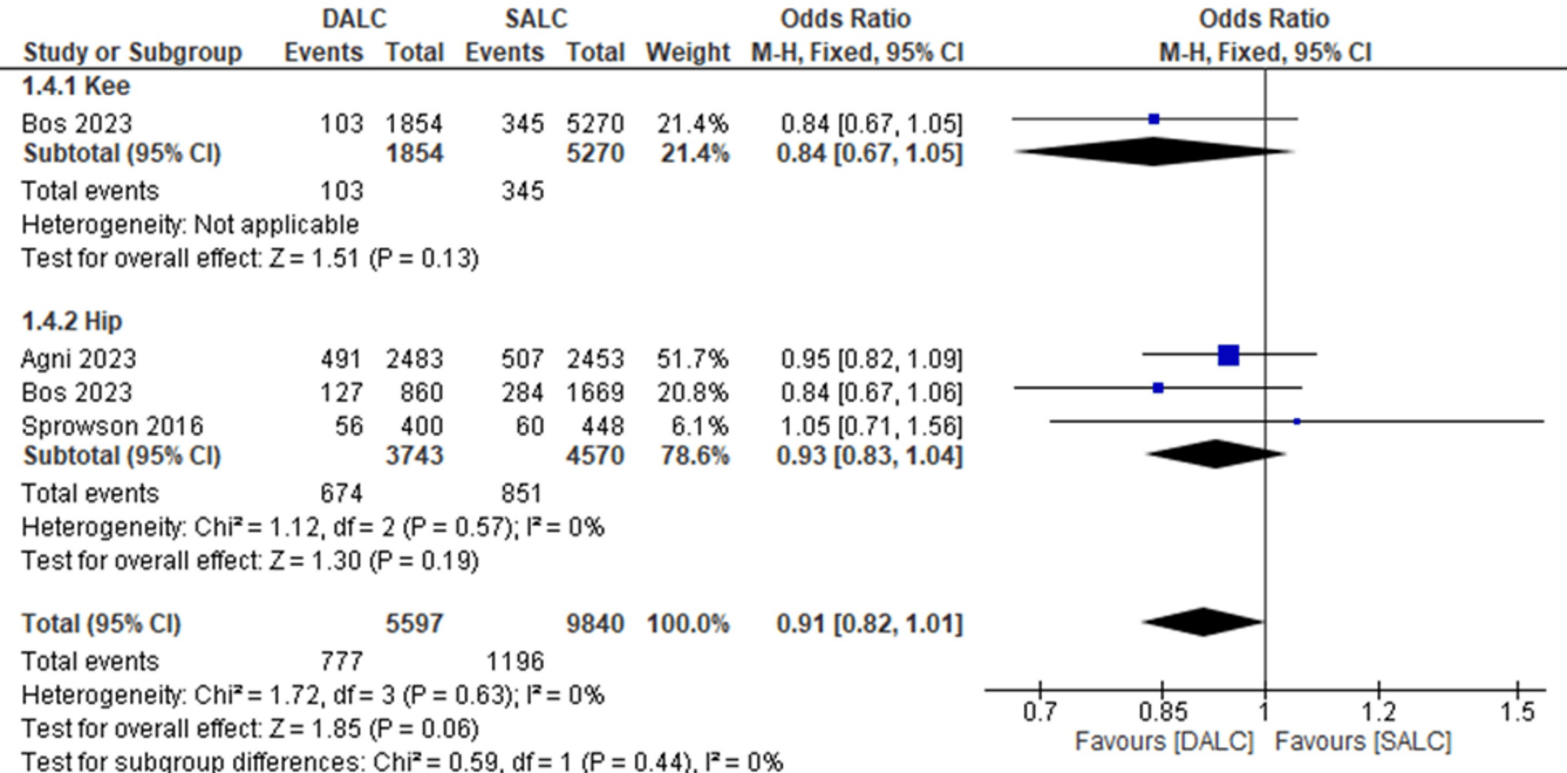
The effects of dual antibiotics loaded cement (DALC) versus single antibiotic-loaded cement (SALC) on the mortality rate. Agni 2023 [16], Bos 2023 [18], Sprowson 2016 [12].

The mortality rate was reported in three studies. In one study in the knee subgroup, the odds ratio (OR) was 0.84 (95% CI (0.67, 1.05), P = 0.13), indicating no significant difference between DALC and SALC in reducing mortality rate. The three studies reported the mortality rates in the hip subgroup, DALC did not reduce mortality rates significantly compared to SALC (OR = 0.93, 95% CI (0.83, 1.04), P = 0.19). There was no heterogeneity (I² = 0%). Overall, there was no significant difference between DALC and SALC in reducing mortality rates (OR = 0.91, 95% CI (0.82, 1.01), P = 0.06). The overall heterogeneity was not significant (I² = 0%). Subgroup analysis showed no significant heterogeneity between knee and hip outcomes (I² = 0%, P = 0.44) (Figure 5).

Discussion

Our systematic review examined dual antibiotic-loaded cement (DALC) efficacy and safety compared with single antibiotic-loaded cement (SALC) in preventing infection during the management of bone fractures; providing a critical inquiry into the relative effectiveness of these two interventions.

While the knee subgroup analysis showed no statistically significant difference between DALC and SALC (OR = 1.21, 95% CI (0.87, 1.70), P = 0.26), the hip subgroup demonstrated a substantial benefit with DALC, reducing infection rates by 36% (OR = 0.64, 95% CI (0.49, 0.83), P = 0.001). The findings align with existing literature, indicating the potential advantages of DALC, especially in high-risk hip arthroplasty cases.

Savage et al. (2019) [20] reported no deep surgical site infection rate with DALC compared to 2.9% in the SALC group, which mirrors our hip subgroup findings. Their data is in favor of using DALC during hip surgeries, especially for patients having total hip replacements because of femur neck fractures. Moreover, Li et al. (2020) [21] found that dual antibiotic prophylaxis (cefazolin and vancomycin) offered better protection against Methicillin-resistant *Staphylococcus aureus *(MRSA) infections, even though the difference in overall SSI rates was not significant. This supports the broader conclusion of our review that DALC's impact is most pronounced when targeting resistant pathogens or in high-risk surgical settings [21-23].

Conversely, Sewick et al. (2020) reported that the use of antibiotic-loaded bone cement (ALBC) did not significantly impact periprosthetic infection rates in primary total knee arthroplasty (TKA), aligning with our knee subgroup findings [24]. This consistency suggests that while DALC may offer an advantage in certain joints and patient populations, the incremental benefit in knee arthroplasty may be limited, possibly due to the inherently lower infection risk or different microbiological profiles between hip and knee surgeries.

The moderate heterogeneity observed in our review (I² = 65% overall, 39% for knees, 52% for hips) underscores the variability in clinical outcomes, surgical techniques, and patient populations across studies. Notably, the significant heterogeneity between knee and hip outcomes (I² = 88.5%, P = 0.003) suggests that joint-specific factors may play a pivotal role in determining the efficacy of DALC.

In our analysis of mortality rates, findings were consistent across both the knee and hip subgroups, indicating no significant difference between the use of dual antibiotic-loaded cement (DALC) and single antibiotic-loaded cement (SALC). Specifically, in the knee subgroup, the odds ratio (OR) was 0.84 (95% CI (0.67, 1.05), P = 0.13), and in the hip subgroup, the OR was 0.93 (95% CI (0.83, 1.04), P = 0.19), both showing no significant reduction in mortality with DALC compared to SALC. Importantly, overall mortality data (OR = 0.91, 95% CI (0.82, 1.01), P = 0.06) further supported the conclusion that DALC does not significantly reduce mortality compared to SALC. These results were accompanied by no significant heterogeneity (I² = 0%), strengthening the robustness of the findings. Our outcomes align with the results reported by Li et al. (2022) [21], who similarly found no significant reduction in mortality rates with DALC versus SALC in primary total knee arthroplasty. This consistency across studies reinforces the notion that while DALC may offer other benefits, it does not appear to confer an advantage in reducing mortality rates compared to SALC in joint arthroplasty procedures.

Our review's strengths are that a large patient population was included and different study designs were considered, which increases the generalizability of the findings. The limitations include the lack of a significant number of randomized controlled trials (RCTs), which weakens the ability to draw causal conclusions. Also, the majority of the included studies were retrospective cohort studies, which are prone to biases such as selection bias and confounding factors. Another important limitation is that we compared the effectiveness of DALC to SALC in revisions regardless of the cause of revision. taking into account that one of the major causes of revision is infection may underestimate the effectiveness of the intervention. In addition, our study did not take into account the difference in the follow-up period, which may affect the outcomes. This is because there were few studies to conduct subgroup analysis to understand the effect of the cause of revision and follow-up period on the patients' outcomes. Also, the effect of the other confounding factors, which may require meta-regression, was beyond the scope of our review.

Additionally, most of the studies reported on both hip and knee surgeries, so the knee surgery subgroup consisted of a comparably small number of studies, which may be less generalizable.

The use of dual antibiotic lavage (DALC) in hip surgeries is supported by evidence, particularly in reducing deep infections. However, due to variability in knee surgery outcomes, further research is needed to explore its benefits. Surgeons should weigh the benefits against the cost and potential adverse reactions. Large-scale randomized controlled trials and cost-effectiveness studies could provide valuable insights for clinical decision-making.

## Conclusions

The study reveals that dual antibiotic-loaded cement (DALC) is more effective in reducing infection rates, especially in hip surgeries, than single antibiotic-loaded cement (SALC). DALC reduces deep surgical site infections and prosthetic joint infections in hip replacement patients. However, it did not significantly improve knee surgeries, suggesting the need for further research on this subgroup. Also, We found no significant difference in re-revision rates or mortality between DALC and SALC. Hence, the clinical advantage of DALC may be limited to reducing infections and no other long-term outcomes, such as reoperations or patient survival. We recommend future large-scale randomized controlled trials focusing on the efficacy, cost-benefit analyses, and long-term effects of dual antibiotic therapies. 
